# V-QBA vs. QBA—How Do Video and Live Analysis Compare for Qualitative Behaviour Assessment?

**DOI:** 10.3389/fvets.2022.832239

**Published:** 2022-03-16

**Authors:** A. S. Cooke, S. M. Mullan, C. Morten, J. Hockenhull, M. R. F. Lee, L. M. Cardenas, M. J. Rivero

**Affiliations:** ^1^Department of Sustainable Agriculture Sciences, Rothamsted Research, North Wyke, Okehampton, United Kingdom; ^2^UCD School of Veterinary Medicine, University College Dublin, Dublin, Ireland; ^3^Bristol Veterinary School, University of Bristol, Bristol, United Kingdom; ^4^Harper Adams University, Edgmond, United Kingdom

**Keywords:** animal welfare, animal behaviour, QBA, qualitative behaviour analysis, cattle, livestock, agriculture, zoology

## Abstract

Animal welfare is an inextricable part of livestock production and sustainability. Assessing welfare, beyond physical indicators of health, is challenging and often relies on qualitative techniques. Behaviour is a key component of welfare to consider and Qualitative Behaviour Assessment (QBA) aims to achieve this by systematically scoring behaviour across specific terms. In recent years, numerous studies have conducted QBA by using video footage, however, the method was not originally developed using video and video QBA (V-QBA) requires validation. Forty live QBAs were conducted, by two assessors, on housed beef cattle to help fill this validation gap. Video was recorded over the assessment period and a second video assessment was conducted. Live and video scores for each term were compared for both correlation and significant difference. Principle component analysis (PCA) was then conducted and correlations and differences between QBA and V-QBA for the first two components were calculated. Of the 20 terms, three were removed due to an overwhelming majority of scores of zero. Of the remaining 17 terms, 12 correlated significantly, and a significant pairwise difference was found for one (“Bored”). QBA and V-QBA results correlated across both PC1 (defined as “arousal”) and PC2 (defined as “mood”). Whilst there was no significant difference between the techniques for PC1, there was for PC2, with V-QBA generally yielding lower scores than QBA. Furthermore, based on PC1 and PC2, corresponding QBA and V-QBA scores were significantly closer than would be expected at random. Results found broad agreement between QBA and V-QBA at both univariate and multivariate levels. However, the lack of absolute agreement and muted V-QBA results for PC2 mean that caution should be taken when implementing V-QBA and that it should ideally be treated independently from live QBA until further evidence is published. Future research should focus on a greater variety of animals, environments, and assessors to address further validation of the method.

## Introduction

Welfare is a central inextricable component of sustainability in livestock systems. Not only is it of moral and ethical importance, but it is also intertwined with animal health, productivity, economics, and environmental impacts (1–3). Consequently, animal welfare can both complement and conflict with other aspects of sustainability (4, 5). There is an increasing consumer demand for high animal welfare, which has led to the commodification of animal welfare and premiums paid for it (6, 7). However, assessing the behavioural and psychological components of animal welfare is difficult and usually a variety of individual measures are employed which may or may not be mathematically integrated to give an overall score [e.g., Welfare Quality® Assessment Protocols (8)].

Qualitative Behaviour Assessments (QBA) have been proposed as a holistic approach to understanding animal welfare (9). The behaviours of individuals or groups of animals are scored systematically across different terms, such as contentedness or uneasiness, on a visual analogue scale, from which a measurement is then taken, and factor reduction applied. This can be done using a fixed list of terms or through free choice profiling. The technique has been applied across a range of animals (including cattle, sheep, pigs, goats, horses, and donkeys) and has been found to correlate with other welfare indicators (10–19). Most recently, the supermarket chain Waitrose and Partners (UK) have begun using QBA to assess the emotional wellbeing of livestock within their supply chain (20), highlighting the commercial application and scalability of QBA.

The QBA methodology was originally designed and validated around live assessments, where the assessor is directly watching the animals *in situ* (9). Since its inception, several studies have used video recordings for QBA (11, 13, 14, 17, 18, 21, 22). Research has indicated that video QBA (V-QBA) results correlate with other welfare indicators (17, 18). Furthermore, Ceballos et al. (23) found that intra-observer and inter-observer reliability was strong for video V-QBA of dairy cattle, results complemented elsewhere (24, 25). However, this was contrasted by Bokkers et al. (21) who assessed inter- and intra-observer reliability of V-QBA, concluding that the technique was “*insufficiently reliable”* due to high intra observer variability. A potential downside of V-QBA is the lack of contextual and sensory information relating to broader welfare, however such information could also be a source of bias in live QBA. Tuyttens et al. (26) found observer bias of students performing video behaviour assessments based on the temperature they believed the barn was. However, Wemelsfelder et al. (27) found that whilst environmental perception may shift results, the effect is likely minor. Live and video assessments also differ in terms of viewpoints. The experiences of live and video assessments are potentially quite different and thus may yield varying results. A live assessment permits the assessor to choose and change their viewing angle, gives an ability to focus, and provides a clearer and more immersive experience than video. However, video provides the benefit of multiple viewpoints at any time.

The primary benefit of V-QBA is the ability to monitor sites at scale, distance, and convenience. This is easier than ever due to the decreasing cost and increasing prevalence of cameras. Notably, they are increasingly common in cattle barns (28–32) and in slaughterhouses, sometimes due to legislation (33, 34). The potential pre-existence of the camera infrastructure further highlights the potential benefits of V-QBA. Other benefits of V-QBA are that any impact of the observer on animals is removed, and, results can be validated at a later date to ensure consistency. However, these benefits can only be truly realised if there is the confidence that V-QBA and QBA have parity and yield comparable results.

To the best of our knowledge, there has been no research conducted to directly and comparatively validate V-QBA against live QBA. Comparisons have however been made in other fields, for example, House et al. (35) and Scaffidi et al. (36) both compared live and video evaluations of surgical procedures and found a good agreement between live and video evaluations. Within the field of animal welfare, the most pertinent study was by Rutherford et al. (18) who showed that V-QBA was sufficient to differentiate between groups of pigs based on their behaviours. Those results show that V-QBA can work as a stand-alone technique, but not how it relates to live QBA and if they can be used in comparative/combined scenarios.

The objective of this study is to provide an insight into just that by directly comparing results derived from V-QBA with those from QBA. This will contribute to the field by providing insight the compatibility of QBA and V-QBA, information crucial for the development of large-scale and remote welfare monitoring programmes.

## Methods

### Experimental Site and Sample Population

The experiment was conducted at the Biotechnology and Biological Sciences Research Council's National Capability, hosted at Rothamsted Research: The North Wyke Farm Platform (Devon, UK) (37, 38). The site rears suckler beef cattle and lambs in a conventional system of outdoor pasture grazing with winter housing (typically November to March/April). This study utilised two similar herds of 30 finishing suckler beef cattle, of Stabiliser (ST) and Stabiliser cross (STX) breeds, all derived from the [North Wyke Farm] same suckler herd and born between 13/01/19 and 14/05/19. The two groups were divided using stratified random sampling based on sex, breed, and weaning weight. The animals entered winter housing on 05/11/19, having been weaned the previous week. The only difference in the management of the two herds was the feed provided to them, both groups received grass (predominately perennial ryegrass; *Lolium perenne*) silage, however one received 3 kg per head of sugar beet (*Beta vulgaris*) pellets incorporated into that. Both groups also had access to an additional maximum of 0.5 kg sugar beet pellet per head via a GreenFeed (C-Lock inc., USA) system, designed to assess methane in breath. Assessing the difference between groups was not an objective of this study. Assessments were split equally across both of these groups.

**Figure 1:**
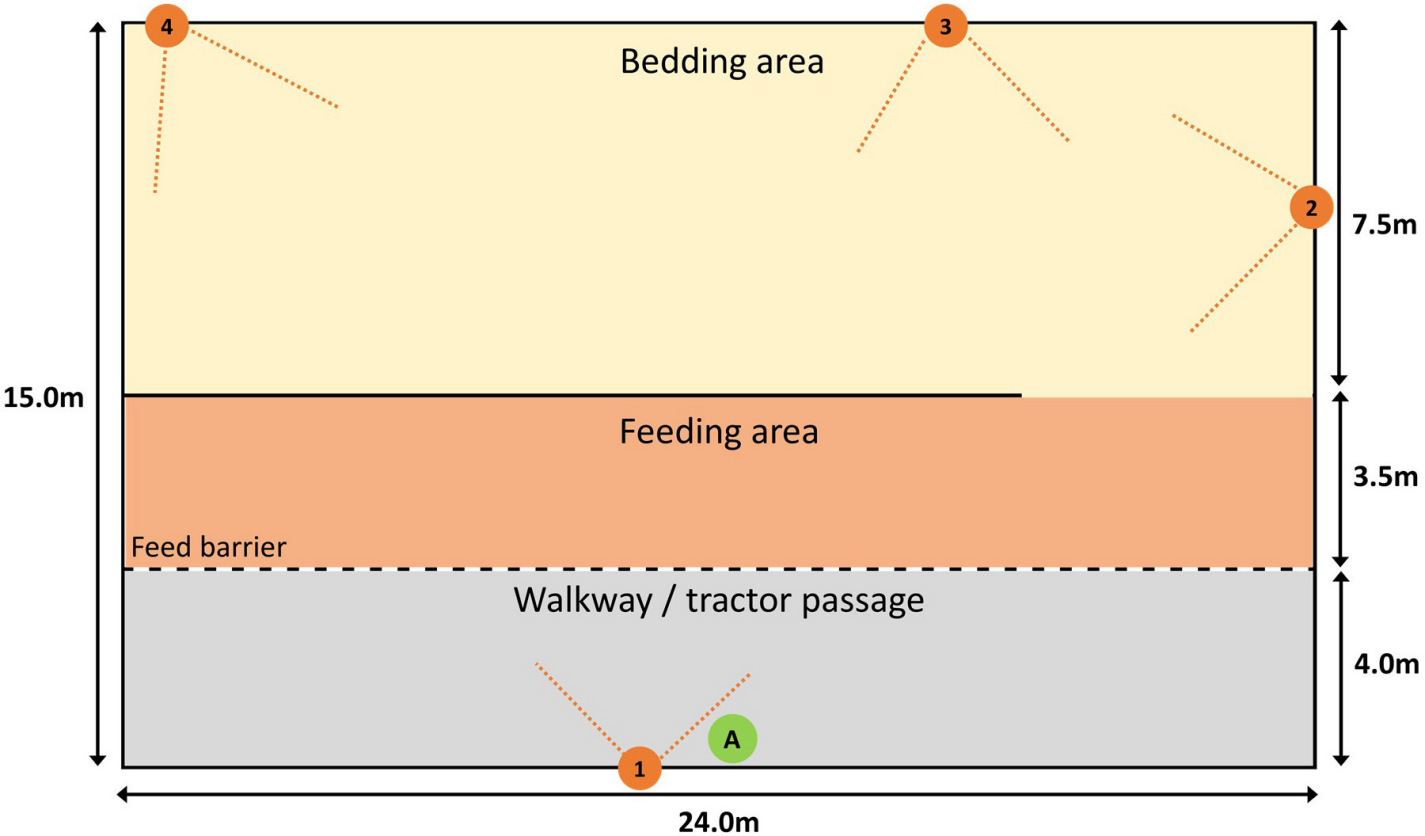
Schematic of the barns, including approximate camera positions and field of view angles (orange). Camera numbers and positions are notated by orange numbered circles (1–4) with the approximate field of view indicated by dashed lines. Cattle had access to the bedding and feed areas, feed was placed in the walkway, the other side of the feeding barriers that separated the feeding area and walkway. The assessor stood by the wall at the bottom of the tractor passage, primarily at the point labelled “A” in the green circle. They were permitted to calmly move along that wall should it be required to gain a better view.

For the duration of the study, both groups were kept in similar adjacent barns of the same specification. The barns were 15 × 48 m internally, including a 4 × 48 m walk/tractor way that cattle had no access to. The study group had half of the available space (11 × 24 m) which included a straw-bedded resting area (7.5 × 24 m) and an eating/drinking area (4.5 × 24 m) (Figure 1).

### QBA Protocol

Each live or video QBA consisted of a 10 min observation period after which assessors immediately scored the group for 20 fixed-terms (Table 1) relating to their behavioural and emotional state. Terms were derived and adapted from Welfare Quality® protocol for cattle (8). Supplementary Material A contains definitions for the terms and information for assessors, which was made available at each QBA. Scoring was conducted by drawing a line on a 125 mm visual analogue scale, the distance along the line was measured to provide a score for that metric. A score of zero represented a complete absence of that characteristic, whilst a score of 125 meant that the state was observed to the greatest realistic extent possible. Supplementary Material B contains a copy of the scoring sheet used in the study.

**Table 1:** Terms for the visual analogue scale of the QBA.

Active	Agitated	Apathetic	Bored
Calm	Content	Distressed	Fearful
Friendly	Frustrated	Happy	Indifferent
Inquisitive	Irritable	Lively	Playful
Positively occupied	Relaxed	Sociable	Uneasy

### Assessments

There was a total of 40 assessment events between 20/11/2019 and 18/03/2020. At each of these events, a live assessment was conducted, with one of two assessors (performing 20 each). Both assessors were agricultural scientists with experience around beef cattle. Crucially, they had received the same training in QBA in October 2019, had similar experience, and had agreed on the term definitions. Thus, inter-observer reliability was high (0.779–0.871, see Supplementary Material C). For each live assessment, they were later (minimum 1 month afterwards) shown video footage of the cattle from the same period, from which they conducted a further assessment on.

Live QBA was conducted as follows: assessors entered the housing facility calmly and stood still. If assessors felt that their entrance evoked an immediate behavioural response in cattle, they waited until they felt confident that that effect had subsided. Assessors observed the animals for 10 min, quietly and with minimal movement so as not to disturb them. Assessment forms were completed immediately after the observation period had finished.

**Figure 2:**
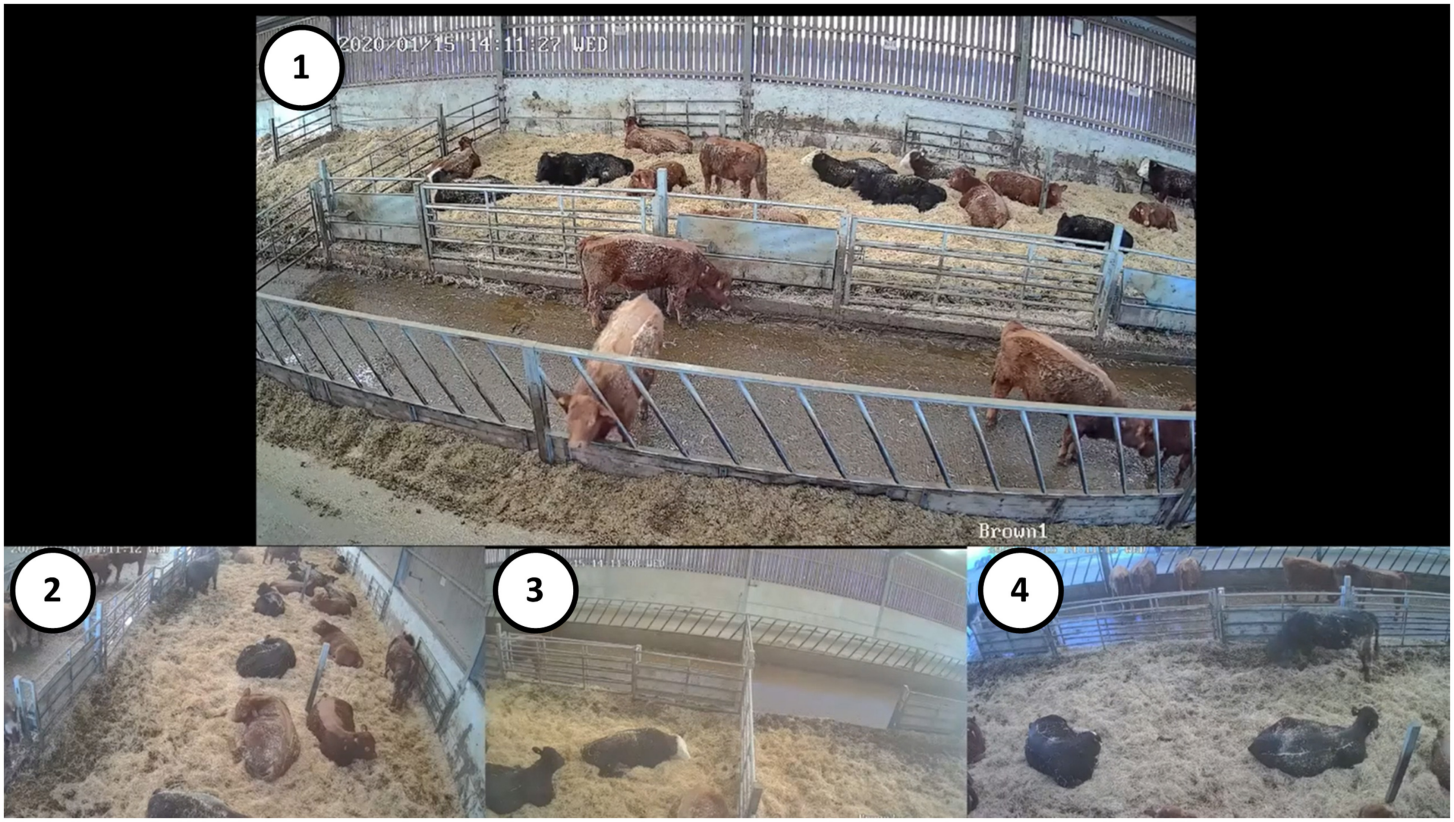
Layout of video collage of the four CCTV cameras installed in each barn, as provided to assessors. Circled numbers have been added for annotation purposes and refer to the cameras from which that video was taken (1–4), as labelled in Figure 1.

For V-QBA, each barn was fitted with four wireless CCTV cameras (1080p, 2.0MP) (Zosi, Zhongshan, China) with fields of view covering the sample population. Cameras were fitted at 4.3 m above ground level (positions 1–4, Figure 1). Video footage was downloaded for the periods covering the live assessments. The four videos for each barn were then formed into a collage (Wondershare Filmora9, Shenzhen, China) to allow all angles to be viewed at once in a single video (Figure 2). Camera 1 provided a viewpoint from a similar position, albeit higher, to that of the assessor and was placed prominently in the video collage. Assessors then watched the video and filled in the QBA assessment form in the same manner as for the live assessments.

### Data Analysis

Results from live and video assessments were compared to identify differences and relationships between results from the two assessment types. No data transformation or outlier removal was performed. Non-parametric methods were used because visual analogue scale data is often considered to be ordinal and, irrespective of that, numerous terms were non-normal (Anderson-Darling Test for Normality).

Paired Sign Tests were applied to each term to identify if there were significant differences in the scores derived from live and video observations. Spearman's rank correlations were then performed for each term to identify if there was an associative relationship between QBA and V-QBA scores. Principle component analysis (PCA) was conducted at the assessment level (one assessment meaning V-QBA or QBA of an observation event). For both PC1 and PC2, values were correlated (Spearman's) between V-QBA and QBA scores from the same event. To further compare the methods for differences, for both PC1 and PC2 paired Sign Tests were conducted to compare V-QBA and QBA scores (negative PC values were transformed to positive values for this [e.g., −0.123 to 0.123]). Based on a 2-dimensional matrix derived from PC1 and PC2, distances between corresponding V-QBA and QBA scores were compared to the distances between all (e.g., including non-corresponding) V-QBA and QBA points using a Mann-Whitney test. A biplot was created combining PC1 and PC2 values (scale transformed from −1.0 to 1.0) to provide a multivariate visualisation of QBA and V-QBA events. Each term was considered a factor, and these were reduced to principal components allowing for each assessment to be summarised to one point. Principal components were defined as mood, arousal, and alertness, this was based on the Welfare Quality Protocol (8) and in-line with descriptions in the literature (17, 39–42).

All statistical analysis and graphing was performed in R (4.04) and R Studio (1.3.959) (43, 44) and utilised packages ggplot2, ggfortify, and Cairo (45–47).

## Results

Results for the terms “Distressed” and “Fearful” were removed from all analyses as they received a score of zero in all instances. “Frustrated” was also removed as it had a high proportion of zeros (65/80). This left 17 remaining terms.

**Figure 3:**
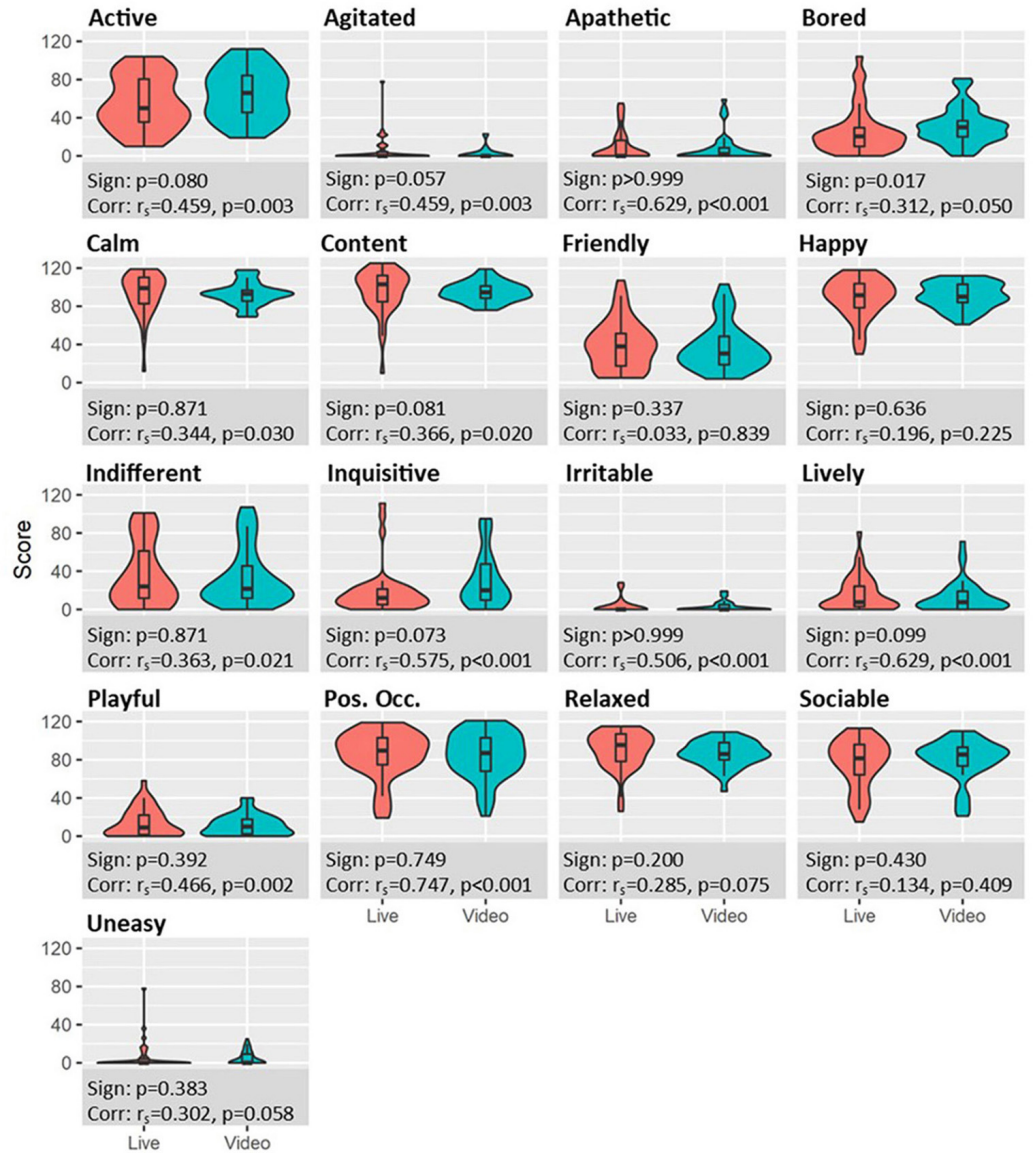
Violin plots of scores for each term, split by live (left, red) and video (right, blue) assessments. Values in footers related to paired Sign tests and Spearman correlation for the respective term.

Pairwise testing found no statistically significant difference in V-QBA and QBA for 16 of the 17 terms. The term “Bored” did yield a significant difference between V-QBA and QBA (median difference +6.5 for V-QBA, *p* = 0.017). Across all terms, there was a high level of visual similarity in the distribution of scores between live and video results, as represented by the shape of the violin plots (Figure 3). Positive and significant correlations were found for 12 of the 17 terms (including for “Bored”), with the strongest correlation being for the term ‘Positively Occupied' (Figure 3).

**Figure 4:**
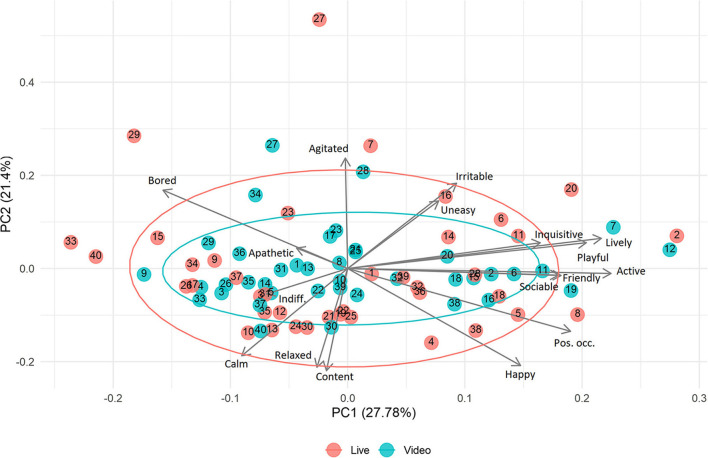
PCA analysis QBA results from live and video assessments, determined by 17 terms. Points with the same number were of corresponding events (e.g., the paired live and video assessments of the same event). Ellipses represent 95% confidence.

**Figure 5:**
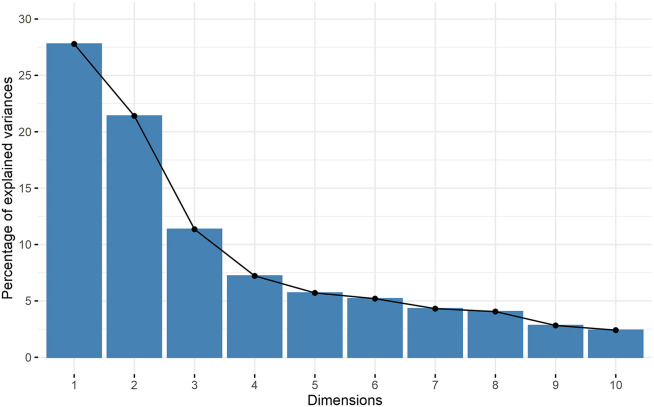
Scree plot showing eigen values of dimensions of PCA, in association with Figure 4.

Principle component analysis showed no clear grouping between V-QBA and QBA scores (Figure 4) (scree plot—Figure 5, loadings table—Table 2). PC1 explained 27.8% of variance, PC2 explained 21.4%, and PC3 11.4%. Loadings showed strong relationships between associated terms. For example, “Bored” and “Apathetic” were closely aligned, as were “Friendly”, “Sociable”, and “Active”. In addition, there were several notable opposing terms, namely “Happy” and “Positively occupied” both opposing “Bored” and “Apathetic”. The predominant terms in PC1 were broadly associated with arousal, whilst for PC2 the terms were associated with mood. PC3 yielded a less clear ordering of terms but appeared may represent alertness. There appeared to be a slightly greater occurrence of QBA points towards the extremities of PC1 and PC2, compared to V-QBA which appeared more centrally, as indicated by confidence ellipses. A statistically significant correlation was found between V-QBA and QBA values for both PC1 (*r*_*s*_ = 0.508, *p* < 0.001) and PC2 (*r*_*s*_ = 0.323, *p* = 0. 0423). There was no significant difference between V-QBA and QBA values for PC1 (*p* = 0.114), however, there was for PC2 (*p* < 0.001), with V-QBA yielding fewer extreme values for PC2. The mean distance between corresponding V-QBA and QBA points was 2.60 (s.d. 1.61) whereas the mean difference between V-QBA points and any random QBA point was 3.52 (s.d. 2.04). This difference was statistically significant (*p* = 0.005).

**Table 2:** Loading values for each term across principal components one (PC1) and two (PC2).

**PC1—Arousal**		**PC2—Mood**		**PC3—Alertness**	
**Terms**	**Value**	**Terms**	**Value**	**Terms**	**Value**
Active	0.383	Agitated	0.404	Indifferent	0.583
Lively	0.369	Irritable	0.312	Apathetic	0.505
Playful	0.347	Bored	0.287	Agitated	0.404
Positively Occupied	0.324	Uneasy	0.248	Content	0.374
Sociable	0.310	Lively	0.110	Inquisitive	0.278
Friendly	0.306	Inquisitive	0.094	Positively Occupied	0.251
Inquisitive	0.280	Playful	0.094	Relaxed	0.192
Happy	0.251	Apathetic	0.075	Bored	0.146
Irritable	0.158	Active	−0.018	Uneasy	0.143
Uneasy	0.132	Friendly	−0.024	Lively	0.106
Agitated	−0.003	Sociable	−0.025	Active	0.069
Content	−0.031	Indifferent	−0.093	Irritable	0.062
Relaxed	−0.045	Positively Occupied	−0.230	Calm	0.029
Apathetic	−0.074	Calm	−0.319	Playful	0.032
Indifferent	−0.118	Happy	−0.355	Happy	−0.032
Calm	−0.154	Relaxed	−0.361	Friendly	−0.118
Bored	−0.268	Content	−0.374	Sociable	−0.225

## Discussion

Across the majority of terms, there was broad agreement between V-QBA and QBA. Whilst three terms were removed due to a high occurrence of zeroes, this itself does highlight further agreement of V-QBA and QBA for those terms, albeit solely at the lower end of the scale. This is supporting evidence that V-QBA is a suitable technique by which to assess housed beef cattle behaviour. However, it is noted that the relationship between V-QBA and QBA scores varies based on which term is being considered. There is some evidence that V-QBA results may provide more conservative responses than QBA.

The lack of significant differences between V-QBA and QBA term scores, across all terms other than “Bored”, provides evidence that there was no notable univariate bias caused by using video as a medium for assessment. There is no clear reason why there was a significant, albeit small, difference for “Bored”. One explanation is that subtle activities (e.g., chewing cud) are less clear over video and thus an animal may appear more bored than if an assessor were present and could see the subtly.

Statistically significant correlations of 12 of 17 terms, combined with the lack of pairwise differences, suggest that V-QBA and QBA are broadly similar in enabling measurement of those terms. Whilst it is not clear why correlations were not present for the five remaining terms, there are some possible explanations for the variability of scoring. Some terms (e.g., “Uneasy”) received consistently low scores with little variation. Whilst the QBA methodology attempts to minimise subjectivity through structured assessment and a continuous visual analogue scale, it is impossible to eliminate it and thus some level of random error and variability remains. The potential impact of this would be greatest on the lower scored terms and this may mask information regarding that term. Correlations for terms were somewhat comparable, in scale and trend, to those reported by Czycholl et al. (48) who studied interobserver reliability of QBA in pigs. Terms that had stronger correlations for interobserver reliability in that study tended to have stronger correlations for inter-technique (V-QBA vs. QBA) within this study. Terms such as “Active”, “Lively” and “Agitated” correlated strongly in both studies, whilst terms such as “Happy”, “Sociable”, and “Bored” yielded some of the weakest correlations in both studies. This suggests that reliability may vary between terms and that interobserver reliability and inter-technique reliability may be similar.

PCA analysis showed no clear clustering of the points, for either technique, along either axis. However, there was an indication that V-QBA results may be slightly muted compared to QBA. This is suggested by the difference in V-QBA and QBA across PC2 and by the smaller confidence ellipse for V-QBA. The exact reason for this is unknown, however it may be the case that, to an observer, a live event is more intense—like watching a sports event, it can be exciting to watch on television, but that excitement is more intense if one is in the stadium itself. Furthermore, the greater proximity of corresponding V-QBA and QBA points, compared to random V-QBA and QBA points, and the correlation between the two techniques for both PC1 and PC2 supports the positive relationship and broad agreement between the two techniques. The relationships between terms, as shown in the PCA as loading factors, were consistent with other research in this area which shows associations between those same or similar terms (13, 49, 50). For example and amongst other similarities, loading plots published by Gutmann et al. (50) showed a positive association between terms “Apathetic”, “Bored”, and “Indifferent” all of which have a negative association with “Sociable” and “Positively Occupied”. A high degree of visual similarity of the distributions, in terms of scale and shape, of scores for all terms, between the techniques, provides evidence that not only supports an absence of bias, but also a level of comparability between the techniques.

There are some limitations to the conclusions that can be drawn from these results. A greater variation and combination of states may have yielded different outcomes, particularly with those associated with negative characteristics (e.g., “Distressed”, “Fearful”), which were not observed frequently within this study. This experiment was conducted on housed finishing cattle and the results may be limited in their validity outside of this context (e.g., grazing cattle, dairy, sheep). Given that V-QBA had to be performed after QBA, this raises the possibility that the QBA assessment may be remembered and biassed the V-QBA assessment. However, the gap between the two was sufficiently long (>1 month) and assessors reported no meaningful recollection of the QBA assessment and thus such a bias is highly unlikely.

### Application

The validity of V-QBA reinforces the opportunity for assessments to be conducted at greater temporal and spatial scales than live assessments otherwise would be. A single researcher could, from one location, conduct analyses of tens of farms worldwide and with observations taken at any time of day or night, providing both scale and resolution simply not possible from live assessments. The real-world implications of this are the potential for more widespread animal welfare assessments, that can improve the welfare component of livestock sustainability [as highlighted by Broom (1)].

In this experiment, the eight cameras and network video recorder (1 Tb) cost approximately £200 (≈$260 USD). Video editing and viewing software are widely available with both paid for and open-source options. Installation and ongoing costs may be offset by the reduction in the need to travel to sites for assessments. Therefore, the financial and technical requirements for implementing V-QBA are relatively low, making it widely available.

In this study, a 10 min assessment period was used to minimise the potential impact that any short-lasting, anomalous, behaviour may have on overall results. However, Rutherford et al. (18) found that 1-minute assessments were sufficient to be able to distinguish two groups of pigs based on behaviour. It is, therefore, possible that a shorter assessment period could be used without significant impacts on the validity of results, but with a positive impact of scale and resources.

Secondary benefits of live assessments, over video, must be considered. Factors such as smells, temperature, wind, and noise, may all act as indicators or drivers of welfare, both positively and negatively. Whilst such factors may be a source of bias when it comes to QBA, they may nevertheless be important. It is therefore important that V-QBA is considered in that context and, where necessary, is part of a broader welfare monitoring programme. This recommendation is in line with comments by Grandin (51) who stated that “*To insure high standards of animal welfare, video methods should never completely replace in-person visits*”.

## Conclusion

This study provides preliminary evidence that V-QBA is a viable alternative to live QBA for housed beef cattle. However, evidence is insufficient at this stage to fully support the interchangeability of the two techniques and suggests that V-QBA may be less sensitive to mood-related behaviours than QBA. It is recommended that QBA and V-QBA are not treated interchangeably and a direct comparison of QBA and V-QBA results could be invalid if differences are not accounted for. Nevertheless, the techniques can complement each other. Whilst comparisons between different sites were not included in this study, this example does highlight that uncertainties can arise through differences in assessment and thus caution should be taken when drawing direct comparisons between sites. Further research, spanning a wider variety of animals, environments, and assessors, would provide important clarity on this. Whilst that uncertainty remains, this is most likely outweighed by the substantial benefits of V-QBA, which could allow for assessments to be conducted at a high scale and resolution, with reduced labour, carbon footprint (travel) and with the ability to repeat assessments.

## Data Availability Statement

The raw data supporting the conclusions of this article will be made available by the authors, without undue reservation.

## Ethics Statement

The animal study was reviewed and approved by Rothamsted Research Animal Welfare Ethical Review Body (AWERB). Written informed consent was obtained from the owners for the participation of their animals in this study.

## Author Contributions

AC: experiment management, experimental design, field work, data collection, data analysis, and manuscript preparation. SM: training, experimental design, data analysis design, and manuscript preparation. CM: field work and data collection. JH: experimental design, training, data analysis design, and manuscript preparation. ML and LC: funding acquisition, project management, and manuscript preparation. MR: funding acquisition, experimental design, and manuscript preparation. All authors contributed to the article and approved the submitted version.

## Funding

The work was funded as part of Rothamsted Research's Institute Strategic Programme Soil to Nutrition (BBS/E/C/000I0320) using the North Wyke Farm Platform (NWFP) National Capability (BBS/E/C/000J0100) both of which are funded by BBSRC.

## Conflict of Interest

The authors declare that the research was conducted in the absence of any commercial or financial relationships that could be construed as a potential conflict of interest.

## Publisher's Note

All claims expressed in this article are solely those of the authors and do not necessarily represent those of their affiliated organizations, or those of the publisher, the editors and the reviewers. Any product that may be evaluated in this article, or claim that may be made by its manufacturer, is not guaranteed or endorsed by the publisher.
