# Supplementary material for: V-QBA vs. QBA—How Do Video and Live Analysis Compare for Qualitative Behaviour Assessment?
Source: Front Vet Sci. 2022 Mar 16;9:832239. doi: 10.3389/fvets.2022.832239 (PMC8966882; doi:10.3389/fvets.2022.832239)
Supplement: Supplementary file 1 [file Data_Sheet_1.pdf]

Supplement A – Welfare Definitions

The following are brief practical definitions of the terms used within the study, written for the purpose of aiding assessment in the field.

ACTIVE – The state of doing something that requires movement, even if that is just chewing or grooming. The more continuous and pronounced the movement, the more active.

AGITATED –Behaviours of any animals being altered due to non-self-factors (e.g. other cattle, human presence).

APATHETIC – More severe than indifferent, animals ignoring / not responding to things that you would expect them to typically react to and/or not noticing stimuli they would be expected to.

BORED – A lack of being positively occupied (though not necessarily negatively occupied) and/or lack of positive stimuli.

CALM – An absence of stressors, fear, and frantic or irrational behaviour.

CONTENT – A state in which the animals are free from stressors and able to perform the behaviours they want to at their own leisure without fear or inhibition.

DISTRESSED – The most 'powerful' term on this list. Distress is an extreme form of unease, manifested in behaviours that may be panicked and irrational. For example, violent bucking, trying to force their way out of pens, highly aggressive non-play fighting

FEARFUL – Applies to fear towards any stimuli (animals, humans, farm vehicles etc) in the immediate environment.

FRIENDLY – Direct positive interaction with other animals. For example, grooming, playing, sharing food.

FRUSTRATED – Struggling or being unable to do something they desire to. For example, if an animal blocked their access to a resource or if feed was out of reach.

HAPPY – More positive than 'content' and calm (e.g. the absence of stressors) – the derivation of pleasure received from the environment and behaviour and the presence and reception of positive stimuli.

INDIFFERENT (negative) – Animals not actively responding to variations in their environment. They may notice and watch, but not react (e.g. get up and move).

INQUISITIVE – The investigation of stimuli and objects (inc. live objects) in their environment.

IRRITABLE – Sensitivity and annoyance by potential stressors (e.g. other animals, their environment disrupting them).

LIVELY – This is stronger than active, all lively animals are active, but not all active animals are lively. The strongest signs of liveliness are actions such as trotting, running, jumping/bucking.

PLAYFUL – Positive interaction with objects or animals for enjoyment purposes.

POSITVELY OCCUPIED – Performing behaviours which are positive to their welfare and general maintenance (e.g. eating, sleeping, drinking).

RELAXED – Feeling comfortable and safe, with minimal stress and unwanted exertion.

SOCIABLE – This is more general than friendly. One example might be if animals chose to feed next to each other, opposed to being spread out, or if they all sleep close together. It is also the synchronicity and cohesion of behaviour.

UNEASY / CALMLESS – Animals seeming to be nervous and not calm. For example, if they are stopping behaviour to keep watch on something or if they are jumpy/sensitive to noise and movement.
