# Supplementary material for: V-QBA vs. QBA—How Do Video and Live Analysis Compare for Qualitative Behaviour Assessment?
Source: Front Vet Sci. 2022 Mar 16;9:832239. doi: 10.3389/fvets.2022.832239 (PMC8966882; doi:10.3389/fvets.2022.832239)
Supplement: Supplementary file 2 [file Data_Sheet_2.pdf]

Date:___/___/_____ Name:

Time:_____________

Group: BROWN GREEN

	MIN	MAX
Active		
Relaxed		
Fearful		
Agitated		
Calm		
Content		
Indifferent		
Frustrated		
Friendly		
Bored		
Playful		
Positively occupied		
Lively		
Inquisitive		
Irritable		
Uneasy / calmless		
Sociable		
Apathetic		
Happy		
Distressed		
