# Supplementary material for: V-QBA vs. QBA—How Do Video and Live Analysis Compare for Qualitative Behaviour Assessment?
Source: Front Vet Sci. 2022 Mar 16;9:832239. doi: 10.3389/fvets.2022.832239 (PMC8966882; doi:10.3389/fvets.2022.832239)
Supplement: Supplementary file 3 [file Data_Sheet_3.pdf]

Supplement C – Inter-observer reliability

1 Introduction

The work presented in the main manuscript is part of a larger programme of welfare research. As such, the two assessors had performed a number of QBAs together and these assessments were used to calculate the inter-rate reliability (IRR) of the assessors.

2 Methods

A total of 44 QBAs were conducted, spanning 22 events, which each assessor conducting a QBA on each event. These assessments took place under the same conditions as QBA in the primary study. Principal component analysis (PCA) was conducted on the resulting scores and Kendall's coefficient of concordance was applied. Test statistics (*W*-values) were interpreted as per Bateson and Martin (2021):

- 0.0-02 – slight agreement
- 0.2-0.4 – low agreement
- 0.4-0.7 – moderate agreement
- 0.7-0.9 – high agreement
- 0.9-1.0 – very high agreement

3 Results

There was a high and statistically significant level of agreement between assessors across PC1, PC2, and PC3.

Table 1 – Summary PCA results showing the % of variance explained by each component. Additionally, the *W*-value and *p*-value, for each PC, from Kendall's coefficient of concordance testing.

	PC1	PC2	PC3
% of variance	30.5	17.6	12.1
W-value	0.871	0.828	0.779
p-value	0.0189	0.0299	0.0493

4 References

Bateson, M., Martin, P., 2021. Measuring Behaviour: An Introductory Guide. Cambridge University Press.
